# The Past, Present, and Future of Economic Evaluations of Precision Medicine at the Committee for Economic Analyses of the Canadian Cancer Trials Group

**DOI:** 10.3390/curroncol28050311

**Published:** 2021-09-21

**Authors:** Kelvin K. W. Chan, Matthew C. Cheung, Dean A. Regier, Annette Hay, Alexander V. Louie, Winson Y. Cheung, Jean-Eric Tarride, Suji Udayakumar, Nicole Mittmann

**Affiliations:** 1 Sunnybrook Health Sciences Centre, Toronto, ON M4N 3M5, Canada; matthew.cheung@sunnybrook.ca (M.C.C.); alexander.louie@sunnybrook.ca (A.V.L.); s4udayak@uwaterloo.ca (S.U.); nicole.mittmann@cadth.ca (N.M.); 2 Canadian Cancer Trials Group, Cancer Research Institute, Queen’s University, Kingston, ON K7L 3N6, Canada; ahay@ctg.queensu.ca (A.H.); winson.cheung@albertahealthservices.ca (W.Y.C.); 3 Canadian Centre for Applied Research in Cancer Control, Canada; dregier@bccrc.ca; 4 School of Population and Public Health, University of British Columbia, Vancouver, BC V6T 1Z3, Canada; 5 Department of Medicine, Queen’s University, Kingston, ON K7L 3N6, Canada; 6 Department of Medical Oncology, Tom Baker Cancer Centre, Calgary, AB T2N 4N2, Canada; 7 Department of Oncology, University of Calgary, Calgary, AB T2N 1N4, Canada; 8 Department of Health Research Methods, Evidence and Impact, McMaster University, Hamilton, ON L8S 4L8, Canada; tarride@mcmaster.ca; 9 Canadian Agency for Drugs and Technologies in Health, Ottawa, ON K1S 5S8, Canada

**Keywords:** economic evaluation, clinical trial, precision medicine

## Abstract

Precision medicine in oncology poses unique challenges to the generation of clinical and economic evidence used for cost-effectiveness analyses that can inform health technology assessment. The conduct of randomized controlled trials for biomarker-specific therapies targeted towards small populations has limitations in regard to feasibility, timeliness, and cost. These limitations result in associated challenges for groups involved in the generation of economic evidence to inform treatment-related decision making, including the Committee of Economic Analysis (CEA) at the Canadian Cancer Trials Group (CCTG). We provide a high-level description and vision about the new paradigm of clinical trial design, generation of economic evidence, and novel approaches to economic evaluations necessary in the space of precision medicine in oncology in Canada. The CEA’s previous approach to precision medicine, including master protocol designs and single-arm studies, is reviewed. Methods and approaches currently under consideration by the CEA and national collaborators, such as the role of real-world and clinical trial evidence in enabling life-cycle assessment of therapies, are explored. Finally, future initiatives being planned in the space of precision medicine at CCTG, such as the incorporation of correlative studies to identify and test high-performing biomarkers in trials, are discussed.

## 1. Introduction

Cost-effectiveness analysis is an important component of health technology assessment (HTA). It is a tool used to inform decision making about what medicine or treatment should be considered for public reimbursement by aiming to determine the “value for money” of an intervention [1]. In other words, it provides economic evaluation evidence of how well medicine or treatment works in relation to how much it costs [2]. Multiple countries and jurisdictions, including Canada, use cost-effectiveness analysis to inform public reimbursement decisions about health technologies, including anti-cancer therapies and companion diagnostics.

Recent advances in cancer therapeutics have changed the treatment landscape rapidly. One such area of advancement is in precision medicine. Precision medicine aims to allow healthcare interventions to be tailored to groups of patients based on their disease susceptibility, diagnostic or prognostic information, or treatment response. While the concept of precision medicine in oncology is promising, it poses unique challenges to generating comparative clinical and economic evidence to prove clinical benefits to patients and efficient use of limited healthcare resources to society. Specifically, the low frequencies of certain biomarkers that are potentially actionable with corresponding matched anti-cancer drugs create new rare disease populations out of what would otherwise be regarded as common cancers. This leads to issues related to the feasibility, timeliness, and cost of conducting traditional randomized controlled trials (RCTs) with adequate power to test whether these precision medicines truly provide clinically meaningful benefits such as improvements in overall survival (OS) and quality of life (QoL) [3].

Advancements in genomics have changed the paradigm of clinical trial design for precision oncology interventions. Master protocols in which multiple parallel drug studies are conducted under a single overarching protocol are increasingly used to test multiple hypotheses. A 2019 systematic review identified 83 master protocols, most in the field of oncology (*N* = 76/83) and ongoing (*N* = 68/83) at the time of the review [4]. Master protocols are often classified as basket trials (*N* = 49/83), umbrella trials (*N* = 18/83), and platform trials (*N* = 16/83) [4].

In oncology, basket trials test therapies for a specific genetic marker regardless of the anatomical location of a tumour and umbrella trials test multiple targeted therapies for a tumour site stratified into subgroups by molecular alteration [4]. Platform trials, which test several interventions against a common control group, allow interventions to enter and exit a trial under a Bayesian decision rule framework based on demonstration of efficacy or futility [4]. These trial designs have their own advantages and limitations. Methods to understand clinical evidence, patient values and need, as well as ethics and implementation, are increasingly being considered as part of health technology assessment and management processes, which are used in parallel with economic evaluations of therapies.

In the absence of high-quality evidence from RCTs, uncertainty and ambiguity in clinical evidence can result from master protocol designs or other single-arm studies. This complicates the generation of economic evidence, both in its design and the resultant magnitude of uncertainty in the cost-effectiveness of precision medicine. The difficulty in generating high-quality and rigorous economic evidence in precision medicine in oncology is a common challenge faced by many groups that are involved in the generation of economic evidence to inform decision making, including our group, the Committee of Economic Analysis (CEA) at the Canadian Cancer Trials Group (CCTG).

The purpose of this discussion paper, commissioned by the CCTG and supported by the Canadian Agency of Drugs and Technologies in Health (CADTH), is to provide a high-level description and vision about the past, present, and future of the generation of economic evidence in the space of precision medicine in oncology in Canada. First, we introduce CEA at CCTG, focusing on its mandate and its previous approach to precision medicine. Then, we discuss methods and approaches that we are presently considering, with our national collaborations. Finally, we describe future initiatives that are planned in the space of precision medicine at CCTG, with some discussion about important unanswered questions for future explorations.

## 2. Canadian Cancer Trials Group and Committee on Economic Analysis

Created in 1980, the mission of the CCTG cooperative oncology group is to develop and conduct clinical trials aimed at improving the treatment and prevention of cancer, with the ultimate goal of reducing morbidity and mortality from the disease [5]. In 1996, the CEA was established in recognition of the increasing importance of economic factors in decision making for the adoption of new therapies [6]. The main role of the CEA is to support the inclusion of an economic evaluation component in a CCTG trial by applying pre-specified criteria that ensure such sub-studies will be impactful for the Canadian context. Criteria that are considered include whether an investigational therapy is potentially costly, if there is modest therapeutic benefit anticipated relative to a large patient population, if there is a high degree of economic impact uncertainty, if the economic evaluation could yield important information in determining routine practice, or, finally, if the economic data will assist future economic evaluations [7].

Economic evaluation has traditionally been embedded within a CCTG RCT by prospectively collecting resource utilization and health utilities for eventual estimations of incremental cost and incremental effectiveness. When necessary, discounting methodologies and adjusting for censoring of data are applied with a final computation of the incremental cost-effectiveness ratio (ICER), including the characterization of uncertainty around incremental cost and effectiveness outcomes.

With advancements in genomics and potential application for oncology therapeutics, the paradigm of clinical trial design in oncology has changed. The CCTG recognizes that these advancements provide opportunities for a personalized approach to treatment. To that end, the CCTG is committed to clinical and economic evaluation of potentially promising new therapies using approaches that are scientifically credible and can inform clinical adoption. Preparation for future rigorous and valid evaluation approaches has become a strategic plan priority for the CCTG, through building upon the expertise and successful evaluations of the past and those presently underway.

Previous economic evaluations conducted within clinical trials in oncology have been published by the CCTG and outline the methods for evaluation of therapies according to tumour type. A historical example of a clinical trial embedded with a pre-planned economic analysis is CO.17, a trial evaluating cetuximab for the treatment of metastatic colorectal cancer in the chemorefractory setting [8]. CO.17 demonstrated that the survival benefit was larger in patients with KRAS wild-type tumours treated with cetuximab, as compared to best supportive care. An economic analysis was pre-planned to determine the cost-effectiveness and cost–utility (i.e., evaluation of incremental cost per quality-adjusted life-year gained) of cetuximab [8]. As such, this was the first large-scale analysis from an oncology cooperative group that collected direct medical resource utilization and health utility values prospectively in an international phase III clinical trial. The analysis was also unique in that data were examined for the overall study cohort and the KRAS wild-type cohort separately, though it is important to note there was no randomization based on biomarker status. The separate correlative economic evaluation revealed differences in the incremental cost-effectiveness and cost-utility ratios in favour of the KRAS wild-type cohort. The economic evaluation of CO.17 demonstrated the potential value of a biomarker-driven approach to oncology treatment by identifying a biomarker-positive subgroup of patients who derive the most clinical benefit while sparing patients in the biomarker-negative subgroup from developing toxicities. This resulted in a better therapeutic index for patients and value for money for the healthcare system [7].

A second CCTG example is the cost-effectiveness analysis carried out within the BR.21 trial, a randomized controlled clinical trial comparing erlotinib, a tyrosine kinase inhibitor, to placebo for the second-line treatment of advanced non-small-cell lung cancer (NSCLC). For this analysis, healthcare utilization costs were prospectively collected to determine mean costs per treatment arm [9]. The analysis demonstrated that the main driver of the cost was erlotinib itself, as the costs of other interventions involved in supportive care such as hospitalizations and clinic visits were similar between treatment arms. In selected clinical and molecular subgroups where erlotinib was found to be more effective, including never smokers and those with an epidermal growth factor receptor (EGFR) mutation, erlotinib was estimated to be a cost-effective treatment [9].

These examples of economic evaluations conducted in the context of RCTs have served to demonstrate the cost-effectiveness of new treatments compared to standard of care and when comparator arms are included in trial designs. In subsequent sections, we will discuss how these types of analyses require novel approaches for the evaluation of precision medicines.

## 3. Challenges of Conducting Economic Evaluation in Era of Precision Medicine

Joint parameter uncertainty in incremental cost and effectiveness drives statistical imprecision, even for trials with large sample sizes. This increases decision uncertainty around comparative value for money of interventions. In the era of precision medicine where sample sizes are smaller due to the focus on a treatment targeted at a specific biomarker within a specific cancer type, there is justified concern that there will be a greater lack of precision in economic evaluations [3]. Phase II studies, sometimes of single-arm design, are used to evaluate safety and efficacy in precision oncology and are often sufficient for regulatory review by Health Canada through the Notice of Compliance with Conditions process [10]. However, in the context of cost-effectiveness analysis reviews by CADTH, phase II data may not meet evidentiary standards [11] due to short time duration for study, use of surrogate endpoints where evidence on OS or QoL is lacking, and finally, a lack of a randomized control group of patients with the same biomarker receiving a treatment comparator (i.e., standard of care), particularly a Canadian-relevant one.

The challenges that are faced in economic evaluation likely require both better methods and more information; however, even with the best possible methods, there is a limit as to how much precision can be achieved. Recognizing this, a tension exists between the pursuit of more precise or perfect information and the opportunity cost of delaying decision making for stakeholders including clinicians, patients, regulators, HTA committees, and payers.

## 4. The Potential of Real-World Evidence (RWE)

### 4.1. Initiatives Exploring the Role of RWE

Real-world evidence utilizes healthcare-system-generated data captured under real-life conditions. Multiple jurisdictions are exploring the role of RWE to examine the external validity of clinical trial evidence and enable life-cycle assessment of pharmaceuticals (i.e., assessment at different points of marker approval) [12]. In Canada, the Canadian Real-world Evidence for Value of Cancer Drugs (CanREValue) Collaboration is one such initiative developing a general framework for the generation and use of RWE to enable life-cycle reassessment to inform ongoing oncology drug funding decisions [13]. In precision medicine, RWE methods to concentrate clinical trial data with systems-generated RWE are being examined by the Canadian Network for Learning Healthcare Systems and Cost-Effective ‘Omics Innovation (CLEO). A goal of CLEO is to use clinical trial and RWE for life-cycle assessment, including for building evidence to support reimbursement decisions, monitoring for appropriate technology implementation, and disinvestment away from low-value technologies [14]. The CEA is exploring the feasibility, acceptability, and benefit of a national harmonized approach to data, in which administrative and clinical trials can be shared and linked [15]. Comparison of highly correspondent patient data from administrative and CO.17 databases found that administrative data had longer follow-up and more complete data for certain cost drivers (e.g., hospitalizations) than clinical trial data, supporting the use of the proposed hybrid approach [16].

### 4.2. Methods to Corroborate the Validity of RWE

While decision makers and research enterprises express enthusiasm regarding the potential of RWE to inform cost-effectiveness, they raise appropriate concerns around its use in HTA deliberative processes [17]. Important among these concerns are improving healthcare systems infrastructure to aid in generating decision-grade data and examining the validity of the analytic methods that underpin RWE.

Decision-grade data are data elements that are generated to be appropriate for use in reimbursement decision making and HTA. Key questions in the use of these data include understanding what specific data are required and how these data can be obtained. Guidelines exist for the reporting of cost-effectiveness studies [18,19], but there is little specificity [20] regarding what data elements are necessary for producing reliable evidence in precision medicine. This limits the impact of both real-world and clinical trial evidence and speaks to the need of defining a core set of data elements. It is particularly important that clinical trialists partner with RWE scientists to develop the core data set. Additionally, given that healthcare systems are heterogeneous in what data are generated, data systems will require improvements on data architecture, data curation that brings together siloed data sources, and resources to enable data scientists to employ natural language processing or artificial intelligence to abstract core elements that are not useable in the current form.

The validity of RWE and its associated analytic methods is particularly salient in precision medicine, where oncology trialists have turned to single-arm studies based on master protocol frameworks. The absence of a randomized control arm introduces selection bias because the effect of an intervention will be confounded by patient characteristics. Real-world evidence scientists respond to selection bias by applying matching methods that aim to minimize or eliminate bias resulting from confounding [21]. Matching methods utilize observational real-world data to generate a synthetic control cohort that can be compared against a single-arm interventional trial cohort. While several methods exist, propensity score matching (PSM) is among the most common [22], with the key assumption that there are no differences between cohorts, conditional on the propensity score [23], a term called ‘ignorability’. To better meet ignorability, RWE scientists in precision medicine have begun examining methods to automate the process of maximizing balance through a supervised machine learning method called ‘genetic matching’ [21,24]. In a recently published study comparing PSM and genetic matching, it was found that genetic matching may outperform PSM for achieving balance, particularly in the context of stratified cohorts [21]. Assuming ignorability is met, applied estimates of an intervention’s comparative effect can be pursued using either nonparametric or parametric analyses, with specific considerations needed for statistical inference (e.g., using bootstrapping techniques) in light of the data preprocessing that occurred through matching analyses [22,25,26,27].

## 5. The Future of Clinical Trials in an Era of Precision Medicine

Though predicting the future is beyond the purview of this group, we can effectively scan the horizon, anticipate, and provide suggestions as to the winds and obstacles ahead, and plan accordingly as we set the sails for the years to come. Indeed, CADTH has recently provided additional guidance recommendations on the economic evaluations of tumour agnostic products [28].

Over the next decade, scientific understanding of tumour biology will continue to expand. New therapeutics that target existing and novel antigens will emerge. New biomarkers will be proposed. Clinical trials will remain and likely become more complex to conduct, as diseases are disaggregated into smaller subgroups, and the interaction between bio-specimen results and clinical outcomes becomes increasingly important. People affected by cancer will wish to have the opportunity to benefit from treatments that may help them. The challenge of paying for the broadening array of diagnostic and therapeutic interventions will persist and, in all likelihood, become more acute. Knowing that precision medicine is here to stay for the indefinite future we, along with many others, are identifying specific ways to embrace these opportunities. Indeed, the experience and proposed approaches of other international settings for the economic evaluation of precision medicine are similar to that of Canada’s [29,30]. Below, we describe themes and select examples that form part of our planned approach to economic evaluations in clinical trials in the era of precision medicine.

### 5.1. Improved Biomarker Incorporation into Clinical Trials

Evaluation of a potential biomarker requires assessment of its analytic validity, clinical validity, and clinical utility. Limitations which currently exist in disease-specific evaluation and implementation are more pronounced in the tumour agnostic setting. A robust, coordinated process is required to identify and test high-performing biomarkers and develop companion diagnostics intended to be adopted as the standard of care. Extensive correlative studies are built into many CCTG clinical trials open to accrual over the next few years; one example is LY.17 [31], a randomized phase II trial of novel combination therapies in relapsed non-Hodgkin lymphoma which incorporates immunohistochemistry, fluorescent in situ hybridization, gene expression profiling, and next-generation whole-exome sequencing. In collaboration with scientist experts in these laboratory aspects, CCTG is developing enhanced parameters to inform the design of clinical trials that incorporate biomarkers. Prospective economic evaluations accompanying these trials will factor in the cost of laboratory testing and the sensitivity and specificity of a given test. A more holistic assessment encompassing impact on both diagnostic testing and drug budgets will provide policymakers with broader information to guide decision making.

### 5.2. Considering New Methodologies

Impressive advances in personalized medicine to date have improved outcomes for patients across multiple cancer types [32,33,34]. Hypotheses to be tested over future years include those exploring how one can maintain the benefit of these breakthroughs at a more affordable price point. Cell-based immunotherapy has enormous potential benefits. Chimeric antigen receptor T (CAR T) cell therapy, now approved by Health Canada and the United States (US) Food and Drug Administration in leukemia, lymphoma, and myeloma, has been transformational with some individuals achieving sustained complete remission where all other treatment modalities had failed [35,36,37]. Mass production is not possible for the current autologous T-cell therapies which are manufactured at highly centralized facilities on a per-patient basis. As a result, CAR T cell therapies are extremely expensive, and current prices for commercial products are USD 373,000–475,000 per dosage [38]. Potential means to reduce cost and expand access to timely cell therapy for patients include a decentralized cell manufacturing model and the development of allogeneic products which may lead to ‘off-the-shelf’ products. These approaches will be tested in clinical trials. Economic considerations of these disruptive approaches require merging of typical health economic methodology with that of the manufacturing industry, incorporating supply chain analysis and value stream mapping.

### 5.3. Data Science Strategy

The future holds a staggering amount of data of different types, from a wide array of sources. Careful consideration is ongoing as to how best to merge clinical trial outcome data safely and effectively with that derived from real-world sources, genomic studies, electronic health records, and ‘wearables’ to yield meaningful information. These data provide more information on health resource utilization and the corresponding costs beyond traditional clinical trial data. Linkage to these data sources potentially allows for more comprehensive costing and longer-term follow-up of patients in a more efficient manner than attempting prospective collection of clinical trials. Those experienced in artificial intelligence, computer science, information technology, privacy regulation, and the patient experience all have key roles to play in the development of harmonized data strategies to support future research.

### 5.4. Collaboration

As alluded to in previous sections, the conduct of clinical trials in the era of precision medicine requires deep and wide collaborations, particularly because it may allow for larger sample sizes, and studies in precision medicine commonly have small sample sizes. This would enable more power and confidence in clinical trial results and corresponding economic evaluations. Indeed, clinical trialists and research groups are planning for greater collaboration. An ambitious example at an advanced stage of development is the North American myeloMATCH (Molecular Analysis for Therapy Choice) [39], the precision medicine initiative of the National Clinical Trials Network. Cooperative groups, supported by the US National Cancer Institute, are designing an array of linked clinical trials to comprehensively study myeloid malignancies. The Tier 1 master screening protocol will provide access to integral laboratory assays with rapid turnaround times for patients across the US and Canada. Results will directly inform assignment to subsequent induction and consolidation treatment protocols. Such an approach enables timely accrual to, and completion of, trials designed for low-frequency sub-populations harboring specific biomarkers.

## 6. Discussion

The work of the CEA within CCTG continues to evolve in the era of modern clinical trial design for precision oncology approaches. Early examples of biomarker-driven economic evaluations demonstrated that enrichment for populations of patients likely to benefit from targeted therapy would result in more favourable cost-effectiveness profiles. Such economic evaluations could still be conducted within the structure of conventional CCTG RCTs, in which prospective resource utilization data and preference elicitation are collected in parallel with clinical outcomes.

In the current era of precision oncology, however, the rapid advances in molecular and genetic diagnostics paired with tailored interventions have resulted in a new paradigm of trial design. In such studies, in which targeted therapies are deployed within biomarker-enriched populations, smaller sample sizes raise the concern of lower precision in ICER estimates [40]. If the ultimate objective in the pursuit of economic evaluation is to support the appraisal, reappraisal, and adoption of treatments, then the combined use of real-world and clinical trial evidence collectively could facilitate life-cycle assessment and comprehensive treatment evaluation in precision oncology.

A new definition of HTA, which emphasizes its use in determining the value of health technology at different points in its lifecycle, has been developed and internationally accepted by the International Network of Agencies for Health Technology Assessment (INAHTA) and Health Technologic Assessment International (HTAi) in 2020 [12]. The life-cycle health technology assessment (LC-HTA) framework is proposed as a mechanism to support the initial clinical trial design warranted to generate a suitable minimal dataset and to determine the subsequent datasets and linkages needed to complete data requirements to support decision making [12,14]. Opportunities and challenges to this approach remain. Firstly, researchers and stakeholders need to ensure the collection of timely decision-grade level evidence. Although the initial clinical trial can provide an early and fulsome dataset inclusive of clinical outcomes, preference-based measures, and health resource utilization data, a time lag is anticipated for the generation of real-world outcome data. At present, new data structures and support to enable curation of patient-level data in real-time are warranted. Furthermore, in Canada, administrative data systems are frequently siloed within jurisdictions. Research groups are reticent to share investigator-led data with those that could use it to confirm clinical- and cost-effectiveness. Across Canada and within jurisdictions, distinct data governance laws and research ethics programs restrict the ability to rapidly share data. In a future state, support for federated data analytics in which data can be shared and analyzed without leaving its jurisdiction of origin could address these barriers. Finally, the interaction between clinical trial co-operative groups, such as CCTG, and groups that generate RWE remains limited at present. A movement towards cooperation and collaboration between these entities is needed to achieve the vision possible with the LC-HTA framework, in which clinical trial design is conducted in parallel with the intended incorporation of RWE.

Clinicians and patients are driving for new interventions based on precision oncology, and new frameworks for addressing their evidence are required. The challenges in our current healthcare systems cannot be ignored, and a realistic approach for moving forward is required. However, opportunities to build infrastructure, architecture, and data curation to promote the novel methods and engage in conversation about frameworks that will be acceptable to trialists, health economists, and decision makers are available.
